# Pharmacokinetics of mirtazapine and its main metabolites after single intravenous and oral administrations in rats at two dose rates

**DOI:** 10.1186/2008-2231-22-13

**Published:** 2014-01-07

**Authors:** Mohammad-Reza Rouini, Hoda Lavasani, Behjat Sheikholeslami, Helen Owen, Mario Giorgi

**Affiliations:** 1 Biopharmaceutics and Pharmacokinetic Division, Department of Pharmaceutics, Faculty of Pharmacy and Drug Design and Development Research Centre, Tehran University of Medical Sciences, Tehran, Iran; 2 School of Veterinary Science, The University of Queensland, Gatton Campus, Gatton, Queensland 4343, Australia; 3 Department of Veterinary Sciences, Veterinary Teaching Hospital, University of Pisa, Via Livornese (lato monte), San Piero a Grado, 56122 Pisa, Italy

**Keywords:** Mirtazapine, Metabolites, Rats, Pharmacokinetics, Bioavailability

## Abstract

**Background:**

Mirtazapine (MRZ) is a human antidepressant drug metabolized to 8-OH mirtazapine (8-OH) and dimethylmirtazapine (DMR) metabolites. Recently, this drug has been proposed as a potential analgesic for use in a multidrug analgesic regime in the context of veterinary medicine. The aim of this study was to assess the pharmacokinetics of MRZ and its metabolites DMR and 8-OH in rats.

**Findings:**

Eighteen fasted, healthy male rats were randomly divided into 3 groups (n = 6). Animals in these groups were respectively administered MRZ at 2 and 10 mg/kg orally and 2 mg/kg intravenously. Plasma MRZ and metabolite concentrations were evaluated by HPLC-FL detection method. After intravenous administration, MRZ was detected in all subjects, while DMR was only detected in three. 8-OH was not detected. After oral administration, MRZ was detected in 3 out of 6 rats treated with 2 mg/kg, it was detected in 6 out of 6 animals in the 10 mg/kg group. DMR was only detectable in the latter group, while 8-OH was not detected in either group. The oral bioavailability was about 7% in both groups.

**Conclusions:**

The plasma concentration of the MRZ metabolite 8-OH was undetectable, and the oral bioavailability of the parental drug was very low.

## Background

Mirtazapine (MRZ) is a tetracyclic antidepressant used mainly in patients affected by depression. Less commonly it is also used as a hypnotic, antiemetic, and appetite stimulant, and for the treatment of anxiety, among other indications [1].

Recently, MRZ use has been extended to veterinary species [2-7]. To the best of the Authors’ knowledge, there is minimal information available on the pharmacokinetics of MRZ in rats [8]. Hence, the aim of this study was to investigate the pharmacokinetics of MRZ and its two metabolites, 8-OH and DMR in rats, after single intravenous (IV) and oral (PO) administrations.

## Methods

The study protocol was approved by the ethics committee of animal studies at Tehran University of Medical Sciences.

*Study one* - Twelve Sprague Dawly male rats, aged 8–10 weeks and weighing 250–300 gr, were used. Animals were randomly assigned to two treatment groups (I and II). Each subject belonging to group I received a single oral dose of 2 mg/kg MRZ using the generic drug in the form of a 30 mg/tablet (Sandoz, Italy). After fasting for 12 h overnight, these rats (n = 6) received the treatment by *gavage*, they remained fasted for 6 h after drug administration. The second group (n = 6) was also treated in the morning following fasting, they were given the same dose of MRZ however this was administered via the intravenous route (achieved by dissolving pure MRZ hydrochloride powder in saline to give a 2 mg/mL solution).

Blood samples for pharmacokinetic analysis (0.5 mL) were collected at intervals of 0, 0.25, 0.5, 0.75, 1, 1.5, 2, 3, 4, 6, 8, 10, 12 and 24 h after MRZ administration via a cannula in the animals’ right jugular vein, and placed in collection tubes containing sodium heparin. The blood samples were centrifuged at 1000 g for 10 min within 30 min of collection, and the harvested plasma was stored at -80°C until analysis.

*Study 2* - Six Sprague Dawly male rats, aged 8–10 months and weighing 250–300 gr, were used. After fasting for 12 h overnight, each subject received a single oral dose of 10 mg/kg MRZ via the oral route in the morning, they remained fasted for 6 h after drug administration. Blood samples were collected as for study one. The chromatographic analysis was performed as described in a previous study [5]. The pharmacokinetic calculations were carried out by WinNonLin v 5.3 (Pharsight Corp,). The parameters predicted from the data were maximum concentration (C_max_) of MRZ and DMR in plasma, and the time required to reach Cmax (T_max_). The area under the concentration *vs.* time curve (AUC_0-∞_) was calculated using the linear trapezoidal rule. Oral bioavailability (F%) was computed from the following formula:

$$F\left(\%\right)=\left(\mathrm{AU}C_{\mathrm{OS}}\times \mathrm{Dos}e_{\mathrm{IV}}\right)/\left(\mathrm{AU}C_{\mathrm{IV}}\times \mathrm{Dos}e_{\mathrm{OS}}\right)\times 100$$

The Shapiro-Wilk test was used to assess the normal distribution of data. The T student test was used to estimate any significant differences between the pharmacokinetics of MRZ and DMR after the two administration routes.

## Findings

### Study one – 2 mg/kg IV and PO administrations

MRZ was quantified in plasma from 0.25 to 24 h or 0.25 to 6 h after IV and PO administrations, respectively. After the IV injection, DMR was quantified in 3 subjects while in the remaining subjects trace levels only were detected (>LOD < LOQ). The AUC ratio of MRZ/DMR was about 26.

DMR was undetectable in the orally administered animals because the concentrations were consistently lower than the LOD. Notably, 8-OH was not detected in any of the plasma samples (regardless of route of administration) either, this is surprising considering the very low LOQ (2 ng/ml) for 8-OH and DMR. Although values were normally distributed according to the Shapiro-Wilk test, wide variations in plasma concentrations were noticed among the rats, especially after PO administration.

After IV administration, all data sets were analyzed using the extended least-squares regression analysis and a two- or a three-compartment open model. This latter analysis was not possible in five rats out of the six. In these rats, statistical analysis of the fit of model to the curves indicated that the data sets were consistent with a two-compartment body model. After PO administration, only 3 out of six MRZ data sets fitted the model. This was a result of the variable and low concentrations of MRZ detected. Hence, the pharmacokinetic data representing this treatment should be used with prudence (Table 1). The oral bioavailability was low (6.6±3.1).

**Table 1:** Main pharmacokinetic parameters of MRZ after IV (2 mg/kg) and PO (2 and 10 mg/kg) administration of MRZ

**Parameters**	**IV (2 mg/kg) (n = 6)**	**PO (2 mg/kg) (n = 3)**	**PO (10 mg/kg) (n = 6)**
**R**^**2**^	0.998 ± 0.002	0.972 ± 0.026	0.967 ± 0.032
**AUC**_**0-∞ **_**(ng h/mL)**	1431 ± 659	95 ± 69	1021 ± 240
**Cl (mL//h/kg)**	1763 ± 1074	315.7 ± 112.3	7522 ± 6695
**VD (mL/kg)**	1972 ± 1295	258 ± 91	43210 ± 18018
**HL alpha (h)**	0.23 ± 0.15	0.10 ± 0.02	2.00 ± 3.16
**HL beta (h)**	1.7 ± 0.9	2.4 ± 0.5	4.8 ± 1.6
**Alpha**	3.60 ± 1.37	2.53 ± 0.91	4.54 ± 1.26
**Beta**	0.63 ± 0.43	0.11 ± 0.04	0.15 ± 0.06
**HL abs (h)**	0.37 ± 0.22	0.09 ± 0.09	0.14 ± 0.04
**T**_**max **_**(h)**	/ ± /	0.24 ± 0.23	0.29 ± 0.10
**C**_**max**_**(h)**	/ ± /	76 ± 39	250 ± 67
**F%**	/ ± /	6.6 ± 3.1	7.0 ± 4.2

R^2^ = correlation coefficient; AUC_0-∞_ = area under the plasma concentration-time curve extrapolated to infinity; Cl = clearance; VD = volume of distribution; HL alpha = distribution half-life; HL beta = disposition half-life; Alpha = distribution constant; Beta = elimination constant; HL abs = absorption half-life; T_max_ = time of peak; C_max_ = peak plasma concentration F% = absolute bioavailability.

The DMR data sets obtained after IV dosing of MRZ, were modeled according to a non compartmental analysis. However, the DMR concentrations were low and variable and the resulting data should be carefully evaluated (Table 2).

**Table 2:** Main pharmacokinetic parameters of DMR after IV (2 mg/kg) and PO (10 mg/kg) administration of MRZ

**Parameters**	**IV (2 mg/kg) (n = 3)**	**PO (10 mg/kg) (n = 6)**
**R2**	0.932 ± 0.044	0.991 ± 0.008
**λz (1/h)**	0.34 ± 0.30	0.04 ± 0.02
**HL λz (h)**	3.64 ± 2.55	19.52 ± 7.95
**T**_**max **_**(h)**	0.83 ± 0.44	1.63 ± 1.60
**C**_**max **_**(ng/mL)**	14.68 ± 10.17	74.58 ± 30.37
**AUC**_**0**_**-**_**∞ **_**(ng h/mL)**	54.50 ± 42.98	506.50 ± 274.12

R^2^ = correlation coefficient; λz = terminal phase rate constant; HL λz = terminal half-life; T_max_ = time of peak; C_max_ = peak plasma concentration AUC_0-∞_ = area under the plasma concentration-time curve extrapolated to infinity.

**Figure 1:**
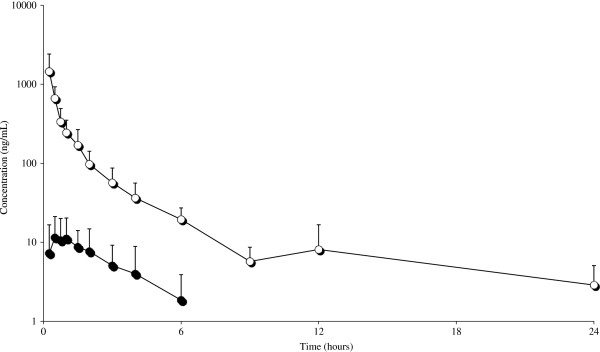
Observed semilogarithmic mean plasma concentrations vs. time curves of MRZ (―○―) [n = 6] and DMR (―●―) [n = 3] following IV single dose administration of MRZ at 2 mg/kg in male rats.

**Figure 2:**
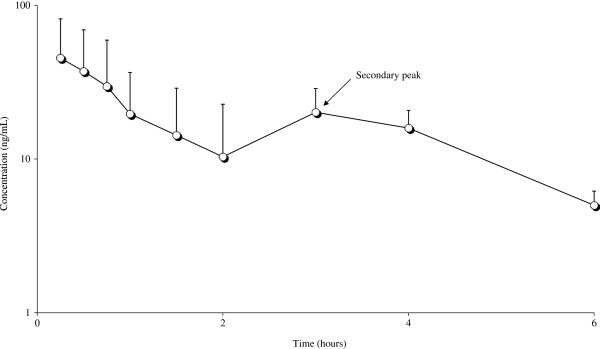
Observed semilogarithmic mean plasma concentrations vs. time curves of MRZ (―○―) [n = 3] following PO single dose administration of MRZ at 2 mg/kg in male rats.

Figures 1 and 2 depict the mean semi logarithmic plasma concentration of MRZ and DMR *vs* time curves respectively, after IV and PO treatments.

### Study two – 10 mg/kg PO administration

**Figure 3:**
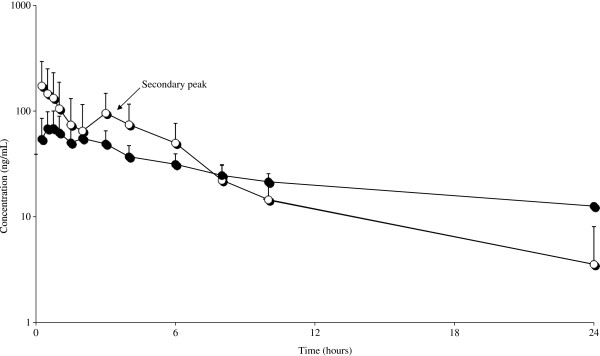
Observed semilogarithmic mean plasma concentrations vs. time curves of MRZ (―○―) [n = 6] and DMR (―●―) [n = 6] following PO single dose administration of MRZ at 10 mg/kg in male rats.

This second study was designed to better describe the pharmacokinetic behavior of MRZ and its metabolites after PO administration. MRZ showed the highest plasma concentrations, followed by DMR, while 8-OH was found in trace concentrations only (< LOQ, > LOD), at all sampling times. The AUC ratio MRZ/DMR value (2), was much higher than for the IV administration. Surprisingly, in the elimination phase 3–5 h after the drug administration, 5 out of 6 subjects showed an unpredicted increase in plasma concentration, for both MRZ and DMR. This effect (a sort of double peak curve) was significant enough to be noticed in the semi-logarithmic mean (n = 6) plasma concentration *vs* time curves shown in Figure 3. The complete set of pharmacokinetic parameters is reported in Table 1. The oral bioavailability was also low (7.0±4.2) with this dose rate.

## Discussion

Recently, there has been movement to investigate potential applications of MRZ in veterinary medicine. In the past few years, MRZ has been tested in cats [3,4], dogs [2] and horses [5]. MRZ caused significant polyphagia in cats [4]. It was hypothesized to be potentially useful for dogs [2] for treatment of anorexia and anxiety-related diseases [9]; exploitation of its antiemetic properties (due to antagonism of 5-HT_3_ receptor) was also considered. In horses, it was suggested as being an analgesic potentially suitable for chronic pain due to its influence on both the noradrenergic and serotonic spinal descending pathways [10]. In rats, MRZ showed significant antinociceptive activity at both the supraspinal and peripheral level [6].

Pharmacokinetics and metabolism have been reported as being highly variable among these species [5]. Although MRZ has been shown to have a good safety profile, caution should be taken in extrapolating doses from other animal species or humans. The low oral bioavailability value found in the current study is noteworthy. To the best of the Authors’ knowledge, this is the first study to report oral bioavailability of MRZ in rats. The oral bioavailability value in humans is 50% [11], but no data is present for any animal species. Since MRZ is mainly administered orally, and its bioavailability might vary considerably among animal species, this parameter should be carefully evaluated in each species. However, even though oral bioavailability in rats is low, the effectiveness of the drug (as an analgesic) has been previously demonstrated [6]. This may suggest that the receptors involved are easily activated, even at low drug concentrations. This speculation could also be supported by the earlier observation of its biphasic activity: its effectiveness was reduced when the dose was increased in rats [6].

Other interesting differences between species have been found in the plasma concentrations of metabolites. 8-OH, which is known to be the predominant metabolite in humans (approximately 40%, [12]) and dogs [2], was not detectable in the present study, mirroring what is reported for horses [5]. The metabolism of MRZ in humans is regulated by phase I biotransformation catalyzed by the enzymes CYP1A2 and CYP2D6 (8-hydroxylation), CYP3A4 [9-11] and probably, CYP3A5 (N-demethylation and N-oxidation) [13]. It is unlikely that the variation in plasma metabolite concentrations between previous human studies and the rat samples in the current study are due to species differences in CYP enzymes. In fact, rat and human CYP2D isoforms share a high sequence identity (>70%) [14]. Additionally, recent studies from Matsubara et al. [15] have identified the new rat CYP3A62 form, and its expression profile is similar to that of human CYP3A4 and rat CYP3A9. The catalytic activities of these enzymes are higher in rats than in humans, but this alone can not account for the large difference in bioavailability. The most plausible explanation is that phase II enzymes account for this difference. It is suspected that the 8-OH metabolite is widely conjugated by glucuronic acid, as several hydroxylated metabolites have shown this metabolic pattern in rats. The rapid elimination of 8-OH as a glucuronide might account for the failure to detect 8-OH in the rat plasma [16]. Further studies are necessary to clarify this issue.

## Conclusion

In conclusion, the present study demonstrates that there are large species differences in MRZ pharmacokinetics. Its oral bioavailability is quite low in rats. The in vivo metabolic pattern appears to be different from that in other animal species and humans.

## Abbreviations

MRZ: Mirtazapine; 8-OH: 8-OH mirtazapine; DMR: Dimethylmirtazapine; HPLC: High pressure liquid chromatography; IV: Intravenous; PO: Oral; CYP: Cytochrome P450.

## Competing interests

None of the authors of this paper has a financial or personal relationship with other people or organizations that could inappropriately influence or bias the content of the paper. The authors declare that they have no competing interests.

## Authors’ contributions

M-RR performed the data analysis and drafted and revised the manuscript. HL and BS participated in the study design, data collection and analysis. HO, contributed in drafting/editing the manuscript. MG computed data, performed statistical analysis and drafted and revised the manuscript. All authors read and approved the final manuscript.
